# A study of the effect of peer-led instruction on the improvement of swimming skills of university students

**DOI:** 10.3389/fpsyg.2026.1768744

**Published:** 2026-07-23

**Authors:** Dan Xie, Wei Song, LiNa Wang, Xiaoyu Lin

**Affiliations:** 1 Civil Aviation University of China, Tianjin, China; 2 Tiangong University, Tianjin, China; 3 Foshan Polytechnic, Foshan, China

**Keywords:** breaststroke, gender differences, peer teaching, physical fitness, swimming instruction, university students

## Abstract

**Background:**

Peer-led instruction has been increasingly adopted in physical education; however, empirical evidence regarding its effectiveness in improving specific aquatic motor skills remains limited, particularly in university swimming education.

**Methods:**

This quasi-experimental study examined the effects of peer-led versus traditional teacher-centred instruction on university students’ 25-m breaststroke kicking performance over a 12-week intervention. A total of 80 s-year university students (19.6 ± 0.8 years; 40 males and 40 females) were included in the final analysis and allocated to either a traditional instruction group (*n* = 40) or a peer-led instruction group (n = 40). The primary outcome was 25-m breaststroke kick time, and secondary outcomes included physical fitness indicators and students’ perceived learning experience. A mixed-design (Group × Time) framework was applied, with effect sizes (Cohen’*s d*) reported to complement significance testing.

**Results:**

Both groups showed significant improvements in breaststroke kicking performance over time. However, the peer-led group demonstrated greater improvement compared with the traditional group (12.5% vs. 8.3%, *t*(78) = 2.56, *p* = 0.02, Cohen’*s d* = 0.57). Significant correlations were observed between several physical fitness indicators and swimming performance (*p* < 0.05). In addition, students in the peer-led group reported significantly higher satisfaction, learning initiative, and perceived skill improvement than those in the traditional group (all *p* < 0.05).

**Conclusion:**

Structured peer-led instruction may be associated with greater improvements in breaststroke kicking performance and more positive learning experiences compared with traditional instruction in university physical education settings. These findings should be interpreted within the limitations of a quasi-experimental design.

## 1 Introduction

Peer-assisted learning and peer teaching models have been widely promoted as student-centred approaches that can enhance learning in school and university physical education. Through structured interaction with same-age or cross-age peers, students may receive more frequent feedback, increased opportunities for practice, and enhanced motivation compared with exclusively teacher-centred instruction (Topping, 2023; Zhou et al., 2023; Zhang and Ke, 2018; Deng, 2019; Luo and Peng, 2015). Theoretical frameworks such as Self-Determination Theory (Deci and Ryan, 2000) and Social Learning Theory (Bandura, 1977) suggest that peer interactions enhance intrinsic motivation, observational learning, and skill acquisition, which is particularly relevant for motor skill development. Motor Learning Theory (Schmidt and Lee, 2011) further emphasizes structured practice and feedback for skill consolidation.

Systematic reviews indicate that peer-led instruction interventions in physical education can improve motor skill performance, engagement, and psychosocial outcomes when properly structured (Topping, 2023; Zhou et al., 2023; Zhang and Ke, 2018; Deng, 2019; Luo and Peng, 2015). Experimental studies have shown that formalised peer-assisted learning approaches can positively influence students’ motivation and skill acquisition (Johnson and Ward, 2001; Ward and Lee, 2005; Hamilton-Hinch et al., 2021; Whipp et al., 2015). Similar benefits of peer-standardised small-group teaching have also been reported in university first-aid training contexts (Lu and Du, 2013).

Within the broader physical activity domain, research on peer-led instruction has examined how dyad composition and gender may shape learning processes. For example, d’Arripe-Longueville et al. (2002) analysed peer interaction modes in swimming skill learning and found that dyad type and gender influenced feedback behaviour and practice opportunities. These findings are further supported by studies highlighting structured peer interaction and teaching design in physical education contexts (Zhu and Dong, 2018; Dong et al., 2018; Li et al., 2017; Sun and Lei, 2013).

In contrast to the growing body of research on peer-led instruction in general PE, studies focusing specifically on swimming pedagogy remain limited. Evaluations of breaststroke instruction in university programs have highlighted issues such as limited instructional time, overreliance on traditional teacher-centred methods, and insufficient individual feedback, all of which may hinder students’ technical improvement and physical development (Pham, 2020; Whipp, 2001; Whipp and Taggart, 2002). Existing studies have highlighted constraints such as limited instructional time, overreliance on traditional teacher-centred methods, and insufficient feedback in university swimming courses (Pham, 2020; Whipp, 2001; Whipp and Taggart, 2002; Liu and Guo, 2021; Hong, 2024; Li et al., 2016; Chen et al., 2019).

Breaststroke kicking performance is influenced by multiple physical and technical factors, including lower-limb power, coordination, and body position. Earlier analytical work on Olympic swimmers in 200-m events also provides relevant evidence on competitive swimming performance characteristics (Shamti et al., 1992). Previous research in competitive swimming has documented sex-based differences in performance across strokes and distances (Li, 2013; Brown et al., 2025). Kinematic analyses further suggest gender-related differences in sprint breaststroke mechanics and performance variables (Matúš et al., 2024). However, most evidence derives from competitive or elite populations, and limited research has examined instructional effects in university physical education settings.

Taken together, existing evidence suggests that peer-led instruction can be an effective pedagogical strategy in physical education (Topping, 2023; Zhou et al., 2023; Jenkinson et al., 2014; Johnson and Ward, 2001; Ward and Lee, 2005; Whipp et al., 2015; Hamilton-Hinch et al., 2021; Zhang and Ke, 2018; Luo and Peng, 2015), and that physical fitness and gender may influence swimming performance (Matúš et al., 2024; Li, 2013). However, there is a lack of integrated research examining peer-led instruction in university swimming education, particularly regarding relationships between physical fitness, gender, and instructional outcomes.

## 2 Materials and methods

### 2.1 Participants and design

Participants were second-year undergraduate students enrolled in compulsory swimming courses at a university in China. A total of 260 students from six intact classes initially volunteered to participate and provided informed consent. The initial cohort included both male and female students with heterogeneous levels of physical fitness and prior swimming experience.

Classes were assigned according to the existing teaching schedule to either a traditional teacher-centred instruction condition (*n* = 121) or a peer-led instruction condition (*n* = 139), resulting in a quasi-experimental design based on intact class grouping.

To ensure eligibility for skill-based instruction and testing, inclusion criteria were applied: (a) adequate water adaptation (ability to perform basic aquatic movements without marked fear), and (b) basic swimming competence (ability to float and perform simple movements). Students who did not meet these criteria or had incomplete pre-test or post-test data were excluded from further analysis.

After eligibility screening, 208 students met the inclusion criteria and had available baseline data for potential inclusion. Because the study was conducted in a natural educational setting without random individual assignment, a complete-case analytical approach was applied within each instructional condition. Students were included in the final analysis only if they had complete pre- and post-test data and met eligibility criteria. The final analytical sample was defined *a priori* as 40 students per condition to ensure balanced group sizes for inferential comparison.

This resulted in a final sample of 80 students, comprising a traditional instruction group (*n* = 40) and a peer-led instruction group (*n* = 40). Baseline characteristics were statistically comparable between groups (Table 1), with no significant differences in physical fitness or initial breaststroke kicking performance.

**Table 1:** Baseline characteristics.

Variable	Traditional	Peer-led	*p*
Age (years)	19.6 ± 0.8	19.7 ± 0.7	0.72
Male/female	20/20	20/20	≥0.99
Height (cm)	176.2 ± 8.4	175.8 ± 8.1	0.83
Body weight (kg)	67.8 ± 13.7	68.1 ± 13.2	0.91
BMI (kg·m^−2^)	21.8 ± 3.7	22.0 ± 3.5	0.79
Vital capacity (mL)	4,398 ± 981	4,472 ± 1,026	0.74
Standing long jump (cm)	219.5 ± 34.6	222.8 ± 36.1	0.68
Sit-and-reach (cm)	16.9 ± 8.7	17.4 ± 8.5	0.81
50-m sprint (s)	7.92 ± 1.12	7.84 ± 1.15	0.76
25-m breaststroke kick (s)	42.3 ± 3.5	41.9 ± 3.8	0.63

Values are presented as mean ± standard deviation unless otherwise indicated. Between-group comparisons were performed using independent-samples *t*-tests for continuous variables and *χ*^2^ tests for sex distribution.

**Figure 1:**
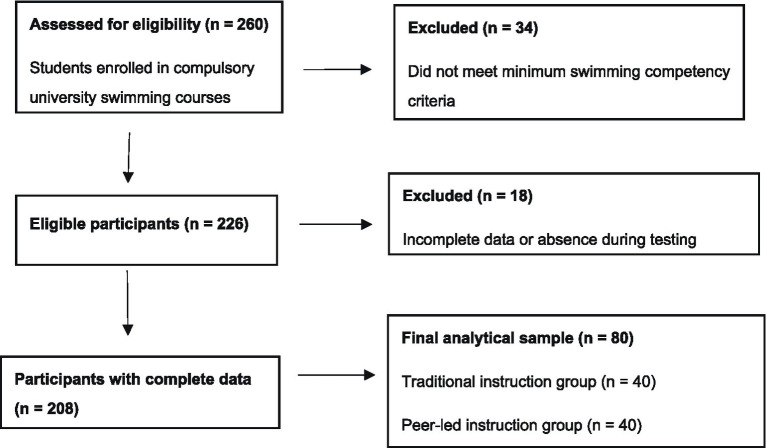
Participant flow diagram for recruitment, screening, exclusion, and final inclusion. Of the 260 students initially assessed, 34 were excluded based on minimum swimming competency criteria. Among the 226 eligible students, 18 were further excluded due to incomplete pre- or post-test data or absence during testing. The final analytical sample included 80 students, comprising 40 in the traditional instruction group and 40 in the peer-led instruction group.

A participant flow diagram summarizing screening, exclusion, eligibility, and final inclusion is presented in Figure 1 to enhance transparency of the recruitment process.

Of the initial 260 students, 34 did not meet swimming competency criteria, and 18 were excluded due to incomplete data or absence during testing. The final analytical sample was derived strictly from participants with complete datasets within intact instructional classes.

It should be noted that this study employed a quasi-experimental design without random individual assignment; therefore, while baseline comparability was statistically confirmed, residual confounding inherent to non-randomized designs may not be fully eliminated and should be considered when interpreting causal inferences.

### 2.2 Measures

#### 2.2.1 Physical fitness tests

Physical fitness was assessed using standardised field tests aligned with the National Student Physical Fitness Standards. The following indicators were recorded:

- Height (stadiometer) and body weight (digital scale), used to calculate body mass index (BMI = kg/m^2^).
- Vital capacity, measured using a spirometer as the maximal exhaled volume after full inhalation.
- Sit-and-reach flexibility, assessed using a standard sit-and-reach box.
- Standing long jump, measured using an infrared timing system.
- 50-m sprint time, recorded using a digital stopwatch.

These variables were used to describe participants’ physical characteristics and to examine associations with swimming performance.

#### 2.2.2 Swimming performance

The primary outcome was 25-m breaststroke kick time (s). Participants performed a breaststroke kick while holding a kickboard, using only lower-limb movement.

Baseline testing was conducted in Week 3 following 2 weeks of water adaptation activities. The intervention lasted 12 weeks, and post-testing was conducted in Week 15. Each participant completed three trials per testing session, and the best performance (lowest time) was recorded as the final score. Lower values indicate better performance.

#### 2.2.3 Student feedback questionnaire

Student perceptions of the instructional methods were assessed using a self-developed questionnaire administered at the end of the course. The instrument included four dimensions: (a) course satisfaction, (b) learning initiative, (c) collaborative communication, and (d) self-rated swimming skill. Items were rated on a five-point Likert scale (1 = very low, 5 = very high), with higher scores indicating more positive evaluations.

The questionnaire was developed based on prior physical education and sport pedagogy research and reviewed by three experts for content validity. A pilot test conducted with students not included in the final sample indicated acceptable internal consistency (Cronbach’*s α* = 0.84).

### 2.3 Instructional procedures

#### 2.3.1 Traditional teacher-centred instruction

In the traditional teaching group, instruction followed a teacher-centred, technique-oriented format. The instructor demonstrated and explained key breaststroke elements (breathing, leg kicks, and arm strokes), followed by whole-class practice focused on repetition and technical refinement. Additional training included aerobic sets and speed drills targeting general swimming fitness. Feedback was primarily provided by the instructor to the whole class or individual students.

#### 2.3.2 Peer-led instruction

In the peer-teaching group, students worked in small groups of four to five. At the beginning of each lesson, the instructor outlined learning objectives and highlighted key technical points. Students then practised within their groups, rotating roles as performer, observer, and feedback provider to support peer learning and technical correction.

Peer tutors were not pre-assigned; instead, leadership roles rotated throughout the intervention, ensuring that all students experienced both instructional and observational responsibilities.

Prior to the intervention, students participated in a structured orientation session (approximately 20 min) led by the instructor, focusing on observation techniques, performance criteria, and principles of constructive feedback. During the intervention, the instructor continuously monitored group activities, provided corrective guidance when necessary, and ensured that peer interactions remained aligned with instructional objectives.

Both groups followed the same 12-week curriculum (one session per week) and received identical technical content. The only difference between groups was the instructional delivery method (teacher-centred vs. peer-led instruction), ensuring equivalence in instructional exposure.

### 2.4 Data analysis

All analyses were conducted using Stata (version 17.0). First, descriptive statistics (mean, standard deviation, and range) were computed for physical fitness variables and 25-m breaststroke kick times at baseline to characterise the sample and assess group comparability. The mixed-design ANOVA was considered the primary inferential model, while *t*-tests were used for supplementary interpretation.

Pearson correlation coefficients were calculated at baseline to examine associations between physical fitness indicators and swimming performance.

All inferential analyses were conducted using two-tailed tests with a significance level of *α* = 0.05.

To evaluate the effects of teaching method on performance, a mixed-design ANOVA (Group × Time) was used as the primary inferential method to assess differences in 25-m breaststroke kick performance over time between the traditional and peer-teaching groups. Paired-samples and independent-samples *t*-tests were conducted as supplementary analyses to describe within-group changes and between-group differences in a more interpretable form.

Gender-stratified analyses were conducted descriptively to explore potential differences in improvement patterns between male and female students within each instructional condition; no formal interaction tests were performed.

For questionnaire data, independent-samples *t*-tests were used to compare satisfaction, learning initiative, collaborative communication, and self-rated swimming ability between groups. Cohen’*s d* was calculated where appropriate to indicate the magnitude of between-group differences.

### 2.5 Ethical considerations

This study was conducted in accordance with the principles of the Declaration of Helsinki and was approved by the Ethics Committee of the Department of Physical Education and Sports, Civil Aviation University of China (reference no. 2025-TWB-001; approval date: 9 March 2025). Participation was voluntary, and all participants were informed about the purpose and procedures of the study. Written informed consent was obtained from all participants prior to data collection.

## 3 Results

### 3.1 Associations between physical fitness and swimming performance

Pearson correlation analysis at baseline showed that several physical fitness indicators were significantly related to 25-m breaststroke kick time (Table 2).

**Table 2:** Correlation matrix between physical fitness variables and 25-m breaststroke kick performance.

Variable	Breaststroke kick time (s)	Height (cm)	Body weight (kg)	Lung capacity (ml)	50-m sprint (s)	Standing long jump (cm)	Sit-and-reach (cm)
Breaststroke kick time	1						
Height	−0.2612	1					
Weight	−0.1908	0.593	1				
Vital capacity	−0.3676	0.5956	0.6394	1			
50-metre sprint	0.4912	−0.3868	−0.1925	−0.4836	1		
Standing long jump	−0.3834	0.4555	0.1703	0.3941	−0.8303	1	
Sit-and-reach	−0.0512	−0.1691	−0.0289	−0.0639	0.2519	−0.1204	1

Height was negatively correlated with breaststroke kick time (*r* = −0.26, *p* = 0.025), suggesting that taller students tended to complete the 25-m kick more quickly. Vital capacity was also negatively correlated with kick time (*r* = −0.37, *p* = 0.001), indicating that students with greater lung capacity generally exhibited better breaststroke kicking performance. Standing long jump distance showed a negative correlation with kick time (*r* = −0.38, *p* = 0.001), implying that greater lower-limb power was associated with faster kicking performance. 50-m sprint time was positively correlated with kick time (*r* = 0.49, *p* < 0.001), meaning that students who were relatively slower in the sprint tended also to be slower in the breaststroke kick, whereas those with shorter sprint times tended to achieve shorter kick times.

Values represent Pearson correlation coefficients. All reported correlations were significant at *p* < 0.05 unless otherwise stated.

Body weight and sit-and-reach flexibility were not significantly associated with breaststroke kick time (both *p* > 0.05). Correlations among fitness variables followed expected patterns (e.g., height with weight, vital capacity with height and weight, 50-m sprint time with standing long jump).

These findings indicate that overall physical fitness-particularly stature, lung capacity and lower-limb power is statistically associated to breaststroke kicking performance in university students.

### 3.2 Effects of teaching method on breaststroke performance

#### 3.2.1 Baseline equivalence

Baseline characteristics are presented in Table 1. No statistically significant between-group differences were observed for age, sex distribution, anthropometric measures, physical fitness indicators, or baseline 25-m breaststroke kick time (all *p* > 0.05), indicating satisfactory comparability between groups prior to the intervention.

#### 3.2.2 Post-intervention performance and improvement

After 12 weeks of instruction (Week 15), both groups demonstrated significant improvement in 25-m breaststroke kick time (Table 3). In the traditional teaching group, mean kick time decreased from 42.3 s to 38.8 s, representing an 8.3% improvement (*p* = 0.03). In the peer-led instruction group, mean kick time decreased from 41.9 s to 36.7 s, representing a 12.5% improvement (*p* = 0.01).

**Table 3:** Post-experiment data comparison.

Teaching method	Number of students	Pre-test (Mean ± SD, s)	Post-test (Mean ± SD, s)	Improvement (%)	*p*-value
Traditional teaching group	40	42.3 ± 3.5	38.8 ± 3.4	8.3%	0.03
Peer-led instruction group	40	41.9 ± 3.8	36.7 ± 3.2	12.5%	0.01

Values are presented as mean ± standard deviation. Improvement (%) was calculated as (pre–post)/pre × 100. Between-group comparisons were performed using independent-samples *t*-tests. Improvement percentages were calculated at group level based on mean values.

An independent-samples *t*-test on improvement scores (post minus pre) indicated that the peer-led instruction group achieved significantly greater improvements than the traditional instruction group (*t*(78) = 2.56, *p* = 0.02; Cohen’*s d* ≈ 0.57). This corresponds to a medium effect size, suggesting that peer-led instruction produced greater improvements in 25-m breaststroke kick time compared with traditional teacher-centred instruction.

### 3.3 Gender differences in improvement

Gender-stratified analyses were conducted descriptively to explore patterns of improvement within male and female students across the two instructional conditions (Table 4).

**Table 4:** Analysis of gender differences on breaststroke kick performance.

Teaching method	Gender	Pre-test (Mean ± SD, s)	Post-test (Mean ± SD, s)	Improvement (%)	*p*-value
Traditional teaching group	Male	34.5 ± 3.2	31.8 ± 3.1	7.8%	>0.05
Peer-led instruction group	Male	34.5 ± 3.0	30.5 ± 2.9	11.6%	>0.05
Traditional teaching group	Female	36 ± 3.4	33.3 ± 3.2	7.5%	<0.05
Peer-led instruction group	Female	36 ± 3.3	31.2 ± 3.0	13.3%	<0.05

Values are presented as mean ± standard deviation. Improvement rate (%) was calculated as (pre-test−post-test)/pre-test × 100. Within-group paired *t*-tests were used for pre–post comparisons, and *p*-values refer to within-group differences. No formal interaction analysis (Group × Time × Gender) was conducted; therefore, gender comparisons are presented descriptively.

Among male students, both the traditional teaching group and the peer-led instruction group showed improvements in 25-m breaststroke kick time (from 34.5 s to 31.8 s and from 34.5 s to 30.5 s, respectively), corresponding to reductions of 7.8 and 11.6%. These changes reflect within-group improvements and were not compared between instructional conditions.

Among female students, the traditional teaching group improved from 36.0 s to 33.3 s (7.5% reduction), while the peer-led instruction group improved from 36.0 s to 31.2 s (13.3% reduction). Within-group changes indicated improvement over time; however, no formal three-way interaction analysis (Group × Time × Gender) was conducted. These findings are reported descriptively and should not be interpreted as evidence of gender-specific intervention effects.

Overall, these descriptive patterns suggest potential differences in improvement trajectories between male and female students; however, these observations remain exploratory and should not be interpreted as evidence of gender-specific effectiveness.

### 3.4 Student perceptions of traditional and peer-led teaching

Quantitative questionnaire results showed that students in the peer-led instruction group reported higher scores across all measured dimensions compared with the traditional teaching group (Table 5).

**Table 5:** Student satisfaction survey.

Feedback dimensions	Traditional teaching group	Peer-led teaching group	*p*-value
Satisfaction score (5-point scale)	3.8 ± 0.5	4.5 ± 0.4	<0.01
Learning initiative score	3.6 ± 0.6	4.4 ± 0.5	<0.01
Collaborative communication score	3.5 ± 0.6	4.6 ± 0.4	<0.01
Swimming skills self-assessment	3.7 ± 0.5	4.3 ± 0.5	<0.05

Satisfaction scores were higher in the peer-led group than in the traditional group (4.5 vs 3.8, *p* < 0.01). Learning initiative scores showed a similar pattern (4.4 vs 3.6, *p* < 0.01). Collaborative communication was rated higher in the peer-led group (4.6 vs 3.5, *p* < 0.01). Self-rated swimming skill was also higher in the peer-led group (4.3 vs 3.7, *p* < 0.05).

Overall, the results suggest that students in the peer-led instruction group reported more positive learning experiences compared with those in the traditional instruction group.

In addition to quantitative data, brief open-ended feedback was collected at the end of the course. Students reported that peer-led lessons were more interactive, facilitated immediate peer feedback for correcting technical errors, and promoted greater cooperation during practice. These qualitative comments were used for descriptive thematic summarization.

## 4 Discussion

### 4.1 Effects of peer-led instruction on breaststroke kick performance

The present study showed that peer-led instruction produced greater improvements in swimming performance compared with traditional instruction, which is consistent with previous findings in physical education research(Topping, 2023; Zhou et al., 2023; Johnson and Ward, 2001; Ward and Lee, 2005; Whipp et al., 2015; Hamilton-Hinch et al., 2021; Zhang and Ke, 2018; Luo and Peng, 2015; Deng, 2019). The present study showed that both traditional teacher-centred instruction and peer-led instruction improved 25-m breaststroke kick performance over a 12-week university swimming course; however, the improvements were significantly greater in the peer-led instruction group. On average, students in the peer-led group reduced their kick time by 12.5%, compared with 8.3% in the traditional group, corresponding to a medium between-group effect size (*t*(78) = 2.56, *p* = 0.02; Cohen’*s d* ≈ 0.57). This finding is consistent with previous research suggesting that structured peer-assisted learning approaches can enhance motor skill acquisition and engagement in physical education contexts (Topping, 2023; Zhou et al., 2023; Johnson and Ward, 2001).

Within the peer-led instruction condition, students were required to observe peers, identify movement errors, and discuss technical solutions in small groups. This structure may have increased active engagement, practice opportunities, and exposure to peer feedback, which in turn could have supported more frequent error detection and correction compared with traditional teacher-centred instruction. However, these effects may also be influenced by other contextual factors, such as variations in individual participation levels or instructor monitoring across sessions.

Taken together, the findings suggest that peer-led instruction may represent an effective pedagogical approach for skill-oriented physical education contexts such as swimming, where repeated practice and timely feedback are important. However, given the quasi-experimental design and single-university sample, the generalisability of these findings should be interpreted with caution.

### 4.2 Physical fitness and breaststroke kicking performance

Physical fitness indicators were significantly associated with 25-m breaststroke kick performance. Height, vital capacity, and standing long jump were negatively associated with kick time, whereas 50-m sprint time was positively associated, suggesting that higher levels of physical fitness may be linked to better swimming performance.

These findings are consistent with previous studies highlighting the relationship between physical fitness and motor performance in physical education contexts (Vasconcellos et al., 2020; Dimitrić et al., 2022; Song et al., 2022; Liu and Guo, 2021). These findings are consistent with previous research highlighting the relationship between physical fitness and motor skill performance in physical education contexts.

These associations may reflect the role of foundational physical capacities in supporting the execution of technically demanding motor tasks. From the perspective of Self-Determination Theory and Motor Learning Theory, higher fitness levels may reduce physical constraints during skill execution, allowing learners to allocate more cognitive resources to movement refinement and error correction.

Overall, these results suggest that individual physical fitness profiles may be an important consideration when designing and implementing both peer-led and traditional instructional approaches in swimming education.

### 4.3 Gender differences in response to teaching method

Gender-stratified analyses descriptively suggested different patterns of improvement between male and female students across instructional conditions. Female students in the peer-led instruction group showed reductions in 25-m breaststroke kick time in descriptive terms across groups; however, no formal interaction analysis (Group × Time × Gender) was conducted. Therefore, these observations should be interpreted as exploratory and descriptive rather than as evidence of gender-specific intervention effects.

Gender-stratified results suggest potential differences in improvement patterns; however, these findings are exploratory due to the absence of formal interaction testing and should be interpreted cautiously in line with previous research on sex-based differences in swimming performance (Matúš et al., 2024; Li, 2013).

From a pedagogical perspective, these findings highlight the importance of considering learner diversity when implementing peer-led instruction, while acknowledging that gender-related interpretations require further confirmation using appropriate interaction-based statistical models.

### 4.4 Student perceptions and learning climate

Beyond performance outcomes, students in the peer-teaching group reported more positive perceptions of the learning experience across all measured dimensions, including satisfaction, learning initiative, collaborative communication, and self-rated swimming ability (all *p* < 0.05). These improvements may be explained by increased peer interaction, feedback frequency, and active engagement during learning tasks, as suggested in previous studies on structured peer-assisted learning (Zhu and Dong, 2018; Dong et al., 2018; Li et al., 2017; Sun and Lei, 2013; Chen et al., 2019).

Qualitative feedback further suggested that students perceived peer-led sessions as more interactive and enjoyable. Participants frequently highlighted the value of immediate peer feedback for identifying and correcting technical errors, as well as the supportive group environment during practice. These descriptions reflect students’ subjective learning experiences rather than objectively measured psychological outcomes.

From a pedagogical perspective, these findings suggest that learning climate may play an important role in skill acquisition in physical education settings. Structured opportunities for peer interaction, feedback exchange, and cooperative practice may contribute to more engaging learning environments, which in turn could support sustained participation in skill-based activities.

### 4.5 Practical implications and limitations

The present findings suggest that structured peer-led instruction may be a useful pedagogical approach for university swimming courses to support students’ technical skill acquisition, engagement, and collaborative learning. From a practical perspective, instructors may consider incorporating peer tutor preparation, structured observation tasks, and guided feedback activities, as well as forming balanced student groups to facilitate interaction during skill practice (Topping, 2023; Zhou et al., 2023).

Several limitations should be acknowledged. First, the quasi-experimental design without random assignment and the criterion-based participant selection may introduce potential selection bias, despite efforts to ensure baseline comparability between groups. Second, the study was conducted in a single university setting, which may limit the generalisability of the findings to other educational contexts. Third, student perceptions were assessed using a self-developed questionnaire, and although preliminary reliability was acceptable, further validation of its psychometric properties is warranted. Fourth, instructor involvement and peer tutor quality were not independently controlled, which may have influenced learning outcomes. Finally, exploratory gender analyses did not include formal interaction testing and should therefore be interpreted cautiously.

Future research may consider using multicentre designs with larger and more diverse samples, validated measurement instruments, and longitudinal follow-up to further examine the robustness and generalisability of peer-led instructional effects. More advanced statistical approaches, such as mixed-effects modelling or repeated-measures ANOVA, may also be applied where appropriate to provide more detailed modelling of change over time.

## 5 Conclusion

The present study suggests that structured peer-led instruction may be associated with greater improvements in 25-m breaststroke kick performance and more positive student learning experiences compared with traditional teacher-centred instruction in a university swimming course.

However, given the quasi-experimental design and the use of intact class groupings, causal interpretations should be made with caution.

Future studies employing randomized controlled designs or advanced longitudinal modelling approaches are recommended to further examine the robustness and generalisability of these findings.

## Data Availability

The original contributions presented in the study are included in the article/supplementary material, further inquiries can be directed to the corresponding author.
